# Polymer coated gold-ferric oxide superparamagnetic nanoparticles for theranostic applications

**DOI:** 10.1186/s12951-018-0405-7

**Published:** 2018-10-13

**Authors:** Muhammad Raisul Abedin, Siddesh Umapathi, Harika Mahendrakar, Tunyaboon Laemthong, Holly Coleman, Denise Muchangi, Santimukul Santra, Manashi Nath, Sutapa Barua

**Affiliations:** 1 Department of Chemical and Biochemical Engineering, Missouri University of Science and Technology, 110 Bertelsmeyer Hall, 1101 N. State Street, Rolla, MO 65409-1230 USA; 2 Department of Chemistry, Missouri University of Science and Technology, Rolla, MO 65409 USA; 3 Department of Chemistry, Pittsburg State University, Pittsburg, KS 66762 USA

**Keywords:** Breast cancer, Combination treatments, Gold-superparamagnetic nanoparticles, Magnetic resonance imaging (MRI), Photothermal treatment, Polymer coating

## Abstract

**Background:**

Engineered inorganic nanoparticles (NPs) are essential components in the development of nanotechnologies. For applications in nanomedicine, particles need to be functionalized to ensure a good dispersibility in biological fluids. In many cases however, functionalization is not sufficient: the particles become either coated by a corona of serum proteins or precipitate out of the solvent. We show that by changing the coating of magnetic iron oxide NPs using poly-l-lysine (PLL) polymer the colloidal stability of the dispersion is improved in aqueous solutions including water, phosphate buffered saline (PBS), PBS with 10% fetal bovine serum (FBS) and cell culture medium, and the internalization of the NPs toward living mammalian cells is profoundly affected.

**Methods:**

A multifunctional magnetic NP is designed to perform a near-infrared (NIR)-responsive remote control photothermal ablation for the treatment of breast cancer. In contrast to the previously reported studies of gold (Au) magnetic (Fe_3_O_4_) core–shell NPs, a Janus-like nanostructure is synthesized with Fe_3_O_4_ NPs decorated with Au resulting in an approximate size of 60 nm mean diameter. The surface of trisoctahedral Au–Fe_3_O_4_ NPs was coated with a positively charged polymer, PLL to deliver the NPs inside cells. The PLL–Au–Fe_3_O_4_ NPs were characterized by transmission electron microscopy (TEM), XRD, FT-IR and dynamic light scattering (DLS). The unique properties of both Au surface plasmon resonance and superparamagnetic moment result in a multimodal platform for use as a nanothermal ablator and also as a magnetic resonance imaging (MRI) contrast agent, respectively. Taking advantage of the photothermal therapy, PLL–Au–Fe_3_O_4_ NPs were incubated with BT-474 and MDA-MB-231 breast cancer cells, investigated for the cytotoxicity and intracellular uptake, and remotely triggered by a NIR laser of ~ 808 nm (1 W/cm^2^ for 10 min).

**Results:**

The PLL coating increased the colloidal stability and robustness of Au–Fe_3_O_4_ NPs (PLL–Au–Fe_3_O_4_) in biological media including cell culture medium, PBS and PBS with 10% fetal bovine serum. It is revealed that no significant (< 10%) cytotoxicity was induced by PLL–Au–Fe_3_O_4_ NPs itself in BT-474 and MDA-MB-231 cells at concentrations up to 100 μg/ml. Brightfield microscopy, fluorescence microscopy and TEM showed significant uptake of PLL–Au–Fe_3_O_4_ NPs by BT-474 and MDA-MB-231 cells. The cells exhibited 40 and 60% inhibition in BT-474 and MDA-MB-231 cell growth, respectively following the internalized NPs were triggered by a photothermal laser using 100 μg/ml PLL–Au–Fe_3_O_4_ NPs. The control cells treated with NPs but without laser showed < 10% cell death compared to no laser treatment control

**Conclusion:**

Combined together, the results demonstrate a new polymer gold superparamagnetic nanostructure that integrates both diagnostics function and photothermal ablation of tumors into a single multimodal nanoplatform exhibiting a significant cancer cell death.

**Electronic supplementary material:**

The online version of this article (10.1186/s12951-018-0405-7) contains supplementary material, which is available to authorized users.

## Background

Assembly of the hyperthermia property of superparamagnetic nanoparticles (NPs) with imaging function into a single nanostructure offers a promising way to dynamically monitor the progress of the disease and enhance therapeutic efficacy. Unlike single-component materials, which usually contain only one unique property of the active ingredient, the ingredients of multicomponent materials offer the possibility of multimodal application of these functional components thereby increasing versatility of these materials [1, 2]. The development of multicomponent materials as theranostic nanosystems has a potential to establish a new therapeutic mode to combine imaging and hyperthermia, which can greatly increase the therapeutic efficacy with real-time monitoring in tissues. Normal tissues and tumors differ only slightly in relaxation time, and hence, cannot propagate proper diagnosis signals during magnetic resonance imaging (MRI) [3]. The development of new MRI contrast agents based on NPs is a fascinating field of research that focuses on improving MRI techniques for the early detection of disease. Superparamagnetic NPs such as ferric oxide (Fe_3_O_4_) have been extensively employed as an MRI contrast agent [4–10], altering the magnetic resonance (MR) signals by reducing the relaxivity through de-phasing of the transverse magnetization [11, 12]. A gold (Au) shell coating may be useful in this regard in order to enhance not only the imaging contrasts for MRI but also the photothermal effect via the plasmon-derived optical resonances of gold shells in the visible and near-infrared region (NIR) [13]. The combination of Au-coated NPs has tremendous potential in improving optical properties, thermal properties, tunable geometry, and imaging contrasts in MRI. As a bifunctional NP, Au–Fe_3_O_4_ can inherit excellent surface chemistry characteristics, unique optical properties (attributed to Au) and superparamagnetic characteristics attributed to Fe_3_O_4_. First of all, the NPs offer size controllability, ranging from few to hundreds of nanometers with different and unique size-dependent properties. Second, the NPs can be easily controlled and manipulated from outside with the help of external magnetic field being operated from a distance. Third, the NPs can provide enhanced contrast in medical imaging that can be used to diagnose the situation efficiently. These characteristics would enhance and broaden the application of these nanoparticles for theranostic applications.

While only a few number of Au and magnetic NPs have been approved for clinical and preclinical trials by the U.S. Food and Drug Administration (FDA) [14–17], toxicity of both coated and uncoated magnetic NPs remain a serious concern [18–21]. Both Au and Fe_3_O_4_ NPs exhibit toxicity to cells via numerous mechanisms including the disruption of the cell membrane, DNA damage, induction of oxidative stress, generation of free radicals, impairment of mitochondrial function and alteration in cell signaling among others [22–24]. The toxicity may not be caused by single NP, but rather due to the aggregation of NPs in biological media and serum [25, 26]. Given the unstable nature of NP formulation, it is hypothesized that an optimum dose of a cationic polymer coating onto Au–Fe_3_O_4_ NPs play a role in determining NP configuration on cellular cytotoxicity as well as direct therapy. Coating the surface of Au–Fe_3_O_4_ NPs with suitable polymers may offer several features: (i) reduced aggregation tendency of the particles; (ii) improved dispersibility; (iii) enhanced colloidal stability; (iv) protected undesirable surface oxidation; (iv) surface conjugation for targeting; (v) decreased cytotoxicity; and (vi) increased intracellular uptake by target cells. A charged polymer coating on the NP surface enhances the electrosteric stabilization by attractive and repulsive forces. Poly-l-lysine (PLL) is a biocompatible polycation that promotes cell adhesion by the presence of amine (–NH_2_) groups [27–29]. PLL has been used to increase blood circulation, improve solubility as well as enhance MRI imaging contrasts of gadolinium imaging chelates [27, 29–32]. Steric stabilization has proven to be an effective method to improve NP colloidal stability in ionic media [33]. A uniform coating of Fe_3_O_4_ NPs with polysaccharides leads to a decrease in saturation magnetization as compared to uncoated particles [34]. This drop in saturation magnetization is undesirable due to poor signaling in MRI imaging. Therefore, there is a need for a stable coating on the surface of Au–Fe_3_O_4_ NPs to prepare a colloidal dispersion in the biological medium and attain high MRI imaging contrasts.

In this study, multifunctional Au–Fe_3_O_4_ NPs were designed for medical imaging and hyperthermal treatments of breast cancer cells. To overcome the limitation of colloidal stability and low dispersity of NPs, a PLL polymeric coating was introduced to the surface of Au–Fe_3_O_4_ as a physical barrier for preventing NP aggregation as well as enhancing their intracellular uptake by breast cancer cells. Our results showed that the PLL coating on Au–Fe_3_O_4_ enhanced it stability in biological fluids such as water, phosphate buffered saline (PBS), cell culture medium and PBS containing 10% fetal bovine serum (FBS). PLL coated Au–Fe_3_O_4_ NPs further enhanced the contrast of MRI imaging signals, exhibited intracellular uptake across the breast cancer cell membrane and decreased the cancer cell viability following photothermal treatment. The novelty of this work is to generate a nanometer-thick PLL layer for the stable dispersion of Au–Fe_3_O_4_ NPs in biological fluids that results in excellent optical, magnetic and therapeutic properties for the cancer treatment.

## Methods

### Synthesis of Au–Fe_3_O_4_ NPs

The Au–Fe_3_O_4_ NPs were synthesized by a one-pot synthesis technique based on the method that has been published by Pariti et al. [35]. Briefly, the NPs were synthesized by injecting 2.5 mM of Fe(CO)_5_, 0.25 mM of HAuCl_4_, 2.5 mM oleic acid, and 2.5 mM of oleylamine into 5 ml of Triton^®^ X-100 at 85 °C. The reaction was conducted for 10 min in a three-neck round bottom flask equipped with a magnetic stir bar and air condenser. The temperature was increased to 300 °C. The product was a black precipitate that was isolated from the reaction mixture by magnetic filtration, followed by ultrasonication, centrifugation, and washing to remove excess Triton^®^ X-100 and any unreacted precursors. The powder collected at the bottom of the centrifuge tube was dried in air. The preparation of Au–Fe_3_O_4_ NPs was confirmed by XRD analysis using a D/max–2400 diffractometer and CuK radiation (λ = 0.1541 nm).

### Polymer coating on Au–Fe_3_O_4_ NPs

To coat the NP surface with PLL, 660 µl of 0.1% ($\frac{w}{v}$) PLL (Sigma Aldrich, MW: 150,000–300,000) was added to 25 mg of Au-Fe_3_O_4_ NPs and incubated for 24 h at room temperature (~ 22°C) under continuous shaking. Unbound PLL was removed by centrifuging at 5000×*g* for 30 min that was repeated twice and measured using the trypan blue assay, spectrophotometry, and a standard curve (Additional file 1: Figure S1a) [36]. The concentration of Au–Fe_3_O_4_ NPs was determined using a standard curve of the NP absorption at 710 nm peak (Additional file 1: Figure S1b). The mass ratio of PLL and NPs were calculated. The presence of PLL coating was confirmed by Fourier Transform Infrared (FT-IR) spectroscopy. The FT-IR spectrometer (Perkin-Elmer) was equipped with an electronic temperature control (ETC) EverGlo IR Source and a deuterated triglycine sulfate (DTGS) detector. FT-IR analysis was carried out for uncoated and PLL coated Au–Fe_3_O_4_ NPs by mixing with potassium bromide (KBr) and a pellet method, after a baseline correction being made with dried KBr, to confirm the compatibility. The pellet was prepared to a pressure of about 5 × 10^6^ Pa, in an evacuated chamber, to produce a clear transparent disc of diameter 2 cm and thickness of 0.2 cm. The spectra were recorded at room temperature (~ 25°C) from 4000 to 400 cm^−1^.

### Nanoparticle characterization

To measure the *d*_*mean*_ of Au–Fe_3_O_4_ and PLL–Au–Fe_3_O_4_ NPs, transmission electron microscope (TEM) images were obtained using a Tecnai F20 at an accelerating voltage of 120 kV. A drop of 10 μl of each NP sample in water was air-dried on carbon-coated copper grids (Ted Pella). Images were recorded and analyzed using ImageJ (version 1.45S, NIH, USA) to calculate *d*_*mean*_ for at least 20 particles. The size distribution of Au–Fe_3_O_4_ and PLL–Au–Fe_3_O_4_ NPs was determined by dynamic light scattering (DLS). The zeta potential was measured via laser Doppler anemometry using a Zetasizer Nano ZS (Malvern Instruments). Samples were prepared in water, PBS, RPMI 1640 cell culture medium and PBS with 10% FBS. Measurements were performed in triplicates and shown as the mean ± standard deviation (S.D.). The X-ray diffraction (XRD) patterns were performed by a Philips X-Pert X-ray powder diffractometer with CuKα (1.5418 Å) radiation from 5 to 90°. The UV–Vis absorption spectra of Au–Fe_3_O_4_ and PLL–Au–Fe_3_O_4_ NPs were measured using a Bio-Tek microplate reader (BioTek Synergy 2) between 400 and 900 nm. Fourier transform infrared (FT-IR) spectroscopy of Au–Fe_3_O_4_ and PLL–Au–Fe_3_O_4_ NPs was carried out using a Perkin Elmer Spectrum GX spectrophotometer. Thermal gravimetric analysis (TGA) was performed using a Q50 thermal gravimetric analyzer (TA instruments). The samples were heated from 25 to 1000 °C at a heating rate of 10 °C/min under N_2_ flow. Results are expressed in weight percent as a function of temperature.

### Spin–spin magnetic relaxation (T2 ms) studies of PLL–Au–Fe_3_O_4_ NPs

To measure the relaxation variables in MRI, Au–Fe_3_O_4_ NPs coated with PLL (PLL–Au–Fe_3_O_4_) were prepared in water at 100 μg/ml concentration. All measurements were conducted at 37 °C using a Bruker’s magnetic relaxometer mq20 (0.47 T, B = 20 MHz) for the spin–spin magnetic relaxation time (T_2_) experiments. The specimens were dispersed in PBS and PBS containing 10% FBS at different Au–Fe_3_O_4_ and PLL–Au–Fe_3_O_4_ NP concentrations (1, 10 and 100 μg/ml). The proton spin–spin magnetic relaxation time, T_2_ was measured as a function of the NP concentration.

### Cytotoxicity of PLL–Au–Fe_3_O_4_ NPs in Breast Cancer Cells

BT-474 and MDA-MB-231 breast cancer cells (ATCC) were cultured at 37 °C and 5% CO_2_ in Hybri-Care (ATCC) and RPMI 1640 (Life Technologies), respectively supplemented with 10% FBS (Corning) and 1% (100 units/ml) Penicillin–Streptomycin (Gibco). Cells in the exponential growth phase were seeded in a 96-well plate at an initial density of 10,000 cells/well and incubated overnight. Different concentrations of PLL–Au–Fe_3_O_4_ NPs (0–500 μg/ml) were added to the cell culture media. After 2 h of incubation, the media was removed, and the cells were washed using PBS followed by re-incubation in cell culture media for additional 72 h. The viable and dead cells were measured using a live–dead (calcein AM; CAM and EthD–1) assay (Invitrogen) and calculated according to the manufacturer’s protocol. The cells without any NP treatments were used as the control. The percentage cell death was calculated by the following equation:

1 $$\% \;{\text{cell}}\;{\text{death}} = \frac{{F_{NPs} - F_{blank} }}{{F_{control} - F_{blank} }} \times 100$$

where F_NPs_ is the Ethd-1 fluorescence of the cells treated with PLL–Au–Fe_3_O_4_ NPs, F_blank_ is the Ethd-1 fluorescence of the cell culture medium alone, and F_control_ is the Ethd-1 fluorescence of the cells without any NP treatments. The concentration at which cell death was less than 10% was identified as a non-toxic dose.

### Intracellular uptake of PLL–Au–Fe_3_O_4_ NPs

Cellular uptake of PLL–Au–Fe_3_O_4_ NPs was observed using a Carl Zeiss Axio observer Z1 research microscope system with Apotome.2 optical structuring device. BT-474 and MDA-MB-231 cells were seeded in 8-well chambers containing 10,000 cells/well and grown until 70% confluence. PLL–Au–Fe_3_O_4_ NPs of 100 μg/ml were added to the wells, incubated for 2 h, and washed using PBS for three times. Live cells were re-incubated with media and imaged on a Zeiss microscope using a 63× objective (water) through phase contrast. The intracellular uptake of PLL–Au–Fe_3_O_4_ NPs by BT-474 and MDA-MB-231 cells were further examined by TEM at a voltage of 80 kV. Briefly, cells were seeded in T25 flasks at a density of 1 × 10^6^ cells/ml. After overnight incubation at 37 °C and 5% CO_2_, the cells were incubated with 100 μg/ml PLL–Au–Fe_3_O_4_ NPs. After incubation for another 24 h, the medium was carefully removed and cells were washed with PBS for three times and fixed at 4 °C for 1 h using glutaraldehyde (2.5% in PBS). Then the cells were further subjected to a sequence of treatments to obtain sections that were subsequently mounted on copper grids before TEM measurements.

### Photothermal treatment of breast cancer cells using PLL–Au–Fe_3_O_4_ NPs

A series of specimens with different PLL–Au–Fe_3_O_4_ NP concentrations (0, 50, 100, 200, 400 and 800 μg/ml) were put into quartz cuvettes and irradiated by an 808 nm laser for 10 min with 1 W/cm^2^ power density. An in situ thermocouple thermometer (Cole-Parmer) was used to record the temperature change. To investigate the in vitro photothermal ablation of breast cancer cells, BT-474 and MDA-MB-231 cells were incubated with 100 μg/ml of PLL–Au–Fe_3_O_4_ NPs in cell culture media for 2 h, washed using PBS (3×) to remove the unbound NPs and re-incubated in the corresponding cell culture media for 72 h. The cells were exposed to NIR light (808 nm pulse laser of 1 W/cm^2^ with a diameter of 6 mm for 10 min) to induce photothermal damage. The following control samples were used: cell culture medium, cells without any treatment, and the cells treated with PLL–Au–Fe_3_O_4_ NPs without any exposure to lasers. After exposure to the NIR light, cells were incubated for an additional 72 h at 37 °C. Cell viability was assessed using CAM and EthD-1 (Invitrogen). To further confirm the in vitro photothermal ablation of breast cancer cells, cytotoxicity following irradiation was also performed using MTT viability assay (Invitrogen). Briefly, BT-474 and MDA-MB-231 cells were seeded in a 96-well plate at a density of 10,000 cells/well. After overnight incubation at 37 °C and 5% CO_2_, cells were incubated with PLL–Au–Fe_3_O_4_ NPs of 0, 50, 100, 200, 400 and 800 μg/ml for 2 h. The medium was replaced with 100 μl of fresh medium. A 10 μl MTT of 5 mg/ml in PBS was added to the medium. Cells were incubated at 37 °C for 4 h for labeling cells with MTT. All medium was removed from the well except 25 μl. A 50 μl DMSO was added to each well for dissolving the insoluble formazan crystals. The absorbance at 540 nm was recorded using a Bio-Tek microplate reader (Synergy 2). Mean and standard deviations of two independent experiments were reported for each sample.

### Statistical analysis

Each experiment was carried out with three independent experiments of at least triplicate measurements. The mean differences and standard deviations were evaluated.

## Results

### Synthesis and characterization of PLL–Au–Fe_3_O_4_ NPs

**Fig. 1:**
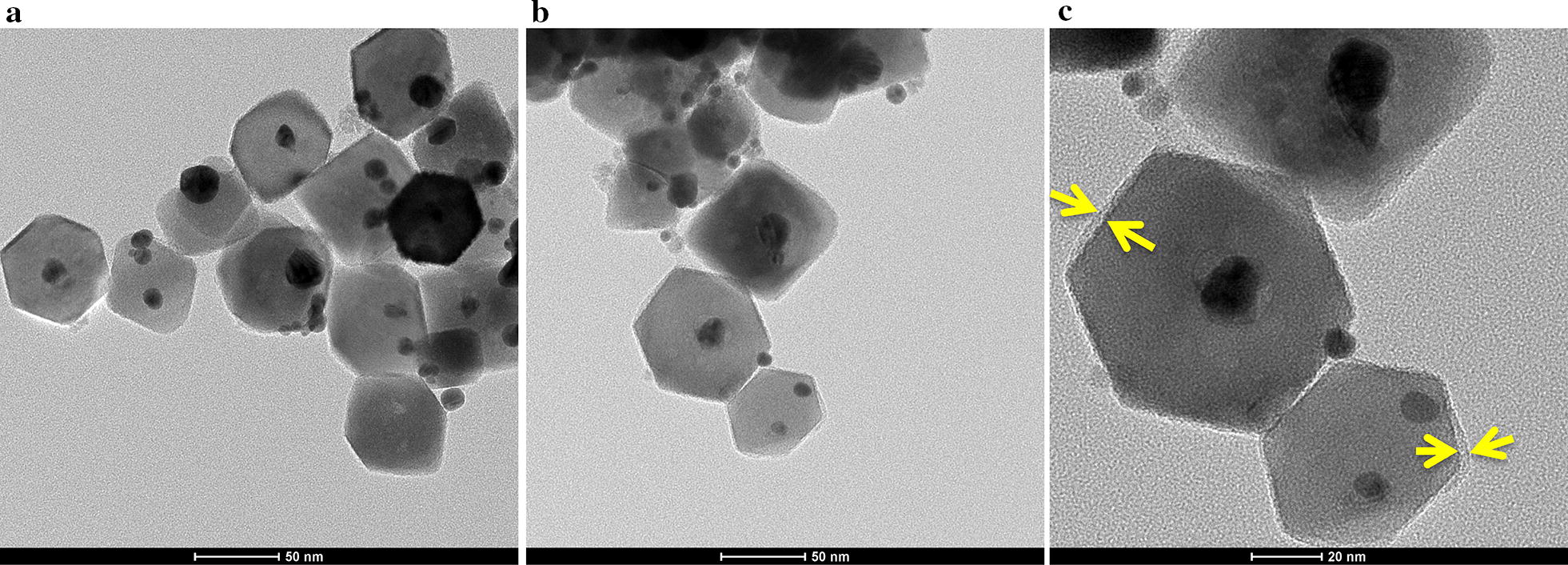
TEM images showing Au–Fe_3_O_4_ NPs **a** without, and **b**, **c** with PLL coating. Scale bar = 50 nm in **a**, **b**, and 20 nm in **c**. The dark black circles indicate Au and the shaded hexagons represent Fe_3_O_4_. Arrows in **c** indicate a thin layer of PLL coating

TEM images revealed that Au–Fe_3_O_4_ NPs were uniform and averaged ~ 55 ± 8.6 nm in diameter (Fig. 1a). The thickness of PLL coating on Au–Fe_3_O_4_ was estimated to be approximately 9 ± 2.5 nm by analyzing the TEM images (Fig. 1b and c). Additional file 1: Table S1 shows the amount of PLL coating on Au–Fe_3_O_4_. The hydrodynamic diameter of Au–Fe_3_O_4_ NPs in water as characterized using DLS showed a wide size distribution of Au–Fe_3_O_4_ dispersions with more than one particle populations and a high polydispersity index (PDI) of 0.856 ± 0.07 (Additional file 1: Figure S2a). The differences in size between TEM and DLS techniques could be explained by NP agglomeration and the presence of small aggregates. The PLL coating decreased the PDI of Au–Fe_3_O_4_ NPs from 0.856 to 0.539 ± 0.03 (Table 1). The hydrodynamic diameters of NPs after PLL coating eliminated the large and small particle aggregates in water, PBS, RPMI 1640 cell culture medium and PBS containing 10% FBS indicating a better colloidal stability (Additional file 1: Figure S2b). The stability of PLL–Au–Fe_3_O_4_ NPs was further proved using a time-resolved video (Additional file 2: Movie S1). As you can see from this video that Au–Fe_3_O_4_ NPs without any coating (left-hand side tube in the video) precipitate down quickly in water while PLL–Au–Fe_3_O_4_ NPs (right-hand side tube) were stably suspended in the black emulsion.

**Table 1:** Characterization of Au–Fe_3_O_4_ before and after PLL coating

Samples	ζ-potential (mV) in various biological medium				
	PDI	Water	PBS	Cell culture medium	PBS + 10% FBS
Au–Fe_3_O_4_	0.856 ± 0.07	− 7.9 ± 0.8	− 12 ± 7	− 3.4 ± 5.2	− 4.8 ± 2.6
PLL–Au–Fe_3_O_4_	0.539 ± 0.03	35 ± 10	26 ± 8.5	15.8 ± 9.2	16 ± 6.8

**Fig. 2:**
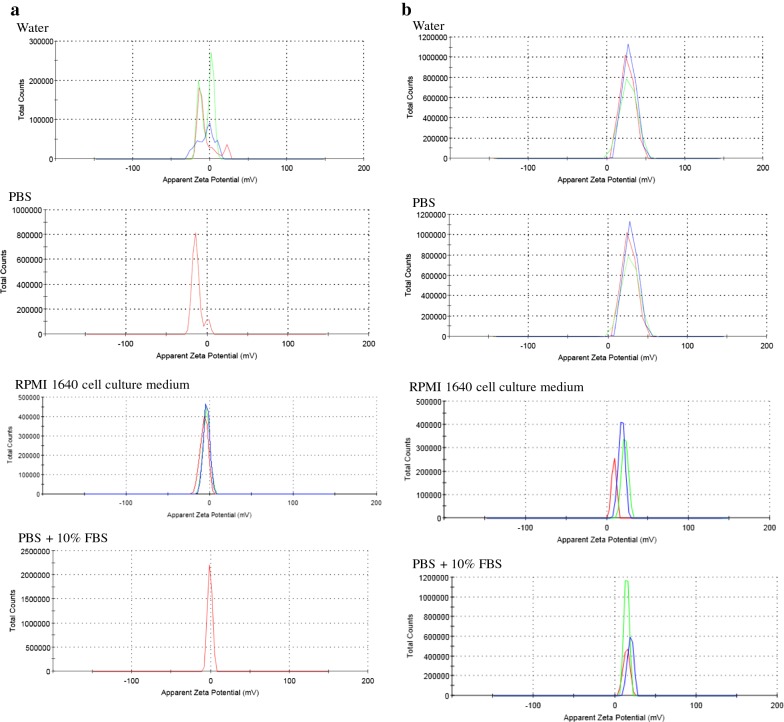
Surface zeta potential of **a** Au–Fe_3_O_4_, and **b** PLL–Au–Fe_3_O_4_ NPs in water, PBS, RPMI 1640 cell culture medium and PBS containing 10% FBS. Three colored (red, green and blue) lines indicate three replicates from three independent experiments. Multiple peaks in Au–Fe_3_O_4_ (**a**) indicate their aggregation behavior that disappears after PLL coating (**b**)

The Au–Fe_3_O_4_ NPs possess a negative surface charge of − 7.9 ± 0.8 mV and − 12 ± 7 mV in water and PBS, respectively as determined by zeta potential (Table 1 and Fig. 2a). The zeta potentials of Au–Fe_3_O_4_ NPs increased in cell culture medium and PBS with 10% FBS indicating pronounced differences in the particle surface chemistry caused by different solvent compositions. The PLL coated Au–Fe_3_O_4_ NPs showed a strong positive charge in water and PBS due to the positive amine groups on the polymer backbone which confirmed the successful grating of PLL polymer on the surface of NPs (Table 1 and Fig. 2b). This further confirms that the PLL coating made the NPs disperse better in aqueous solutions and as a result no agglomeration on storage. Particles that are surface engineered with amine functional groups like PLL has been reported to the effective release of encapsulated molecules from endosomal compartments [37].

**Fig. 3:**
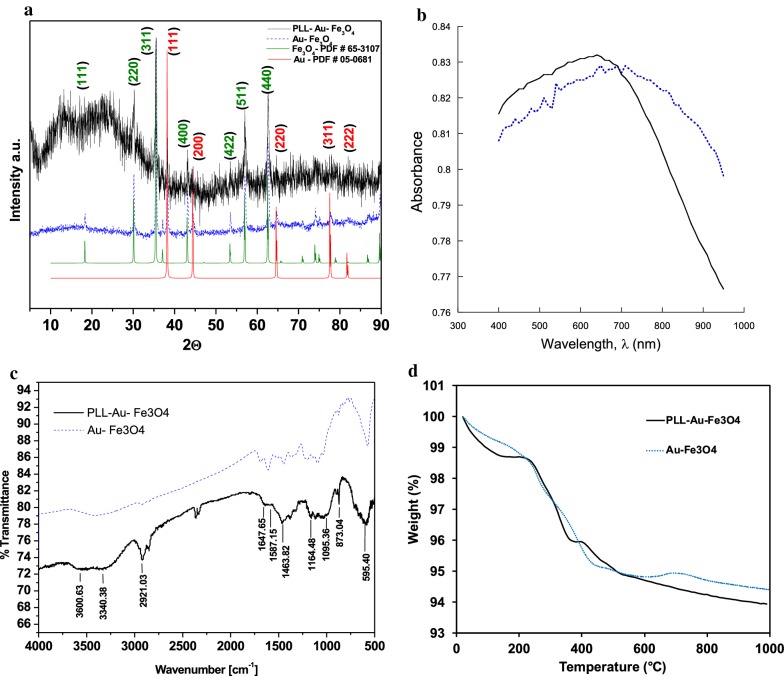
**a** XRD patterns; **b** Absorption spectra (solid line: PLL–Au–Fe_3_O_4_ NPs and dotted line: Au–Fe_3_O_4_ NPs); **c** FT-IR characterization and **d** TGA curves of Au–Fe_3_O_4_ and PLL–Au–Fe_3_O_4_ NPs

The crystal structures of Au–Fe_3_O_4_ and PLL–Au–Fe_3_O_4_ NPs were recorded by the X-ray diffraction (XRD), as shown in Fig. 3a. The XRD pattern of Fe_3_O_4_ shows characteristic peaks at (111), (220), (311), (400), (422), (511) and (440), and (111), (200), (220), (311), (222) peaks for Au which is in agreement to the controls of Fe_3_O_4_–PDF # 65–3107 and Au–PDF # 05–0681, respectively. XRD pattern contains no impurity peak indicating the high purity of Au and Fe_3_O_4_ samples and perfect phase transformation. The XRD pattern of PLL–Au–Fe_3_O_4_ NPs has similar diffraction peaks as those of Au–Fe_3_O_4_ NPs with more peak to noise ratio which could be attributed to PLL polymeric coating.

To confirm the successful synthesis of NPs, UV–Vis absorption and FT-IR spectra were measured. A complete spectra analysis of Au–Fe_3_O_4_ NPs shows a broad surface plasmon absorption with an absorption maximum at around 710 nm (λ_max_) (Fig. 3b; dotted line) [38]. The PLL coating showed an absorption shift to lower wavelengths with an absorption maximum at 640 nm (Fig. 3b; solid line). This explains the enhanced dispersion of PLL–Au–Fe_3_O_4_ NPs in the aqueous phase by redistributing agglomerated Au–Fe_3_O_4_ NPs.

FT-IR spectra were used to study the transformation of Au–Fe_3_O_4_ and PLL–Au–Fe_3_O_4_ NP compositions (Fig. 3c). As shown in Fig. 3c, the strong absorption peak at about 590 cm^−1^ in the FT-IR of Au–Fe_3_O_4_ originates from Fe–O stretching of the Fe_3_O_4_ core. The bare Au–Fe_3_O_4_ spectrum contains a peak at 1630 cm^−1^ due to water physisorbed on the iron oxide surface and a broad band around 3400 cm^−1^ due to surface hydroxyl groups (Fe–OH). For PLL–Au–Fe_3_O_4_ NPs, new and broad absorption bands were observed in the range of 3340 and 3600 cm^−1^, which were attributed to amine N–H stretching and O–H stretching, respectively. The peaks at 1647 and 1587 cm^−1^ attributed to the stretching vibration of C=O from the PLL coating.

To quantify the amount of PLL on the surface of PLL–Au–Fe_3_O_4_ NPs, TGA was performed (Fig. 3d). The PLL coating results in a weight loss of 7.4% between 100 and 650 °C when compared with the uncoated particles (Au–Fe_3_O_4_). No change in PLL–Au–Fe_3_O_4_ mass was observed above 850 °C. Combinedly these findings suggest a successful surface functionalization of Au–Fe_3_O_4_ NPs by PLL polymer coating.

### Determination of T_2_ magnetic relaxation of Au–Fe_3_O_4_ and PLL–Au–Fe_3_O_4_ NPs using magnetic relaxometer

It is important to determine the overall magnetic properties of Au–Fe_3_O_4_ and PLL–Au–Fe_3_O_4_ NPs for cancer imaging by MRI technique. It is expected that the synthesize Fe_3_O_4_ nanocomposites will be MR active as they are superparamagnetic in nature. The spin–spin MR times (T_2_ ms) were analyzed in order to evaluate the magnetic properties of Au–Fe_3_O_4_ with and without the PLL coating (Additional file 1: Figure S3). Generally, the spin–spin magnetic relaxation of water protons changes in the presence of magnetic materials in the solution, such as synthesized Au–Fe_3_O_4_ NPs. This leads to enhance the magnetic property of the solution. Fe_3_O_4_ NPs are generally used for MR contrast agents because of their capacity to shorten the T_2_ relaxation time of their surrounding protons. As can be clearly seen in Additional file 1: Figure S3, T_2_ was decreased gradually with the increase in NP concentration. The T_2_ relaxation time of PLL–Au–Fe_3_O_4_ NPs was higher than that of Au–Fe_3_O_4_ NPs due to the fact that the polymer coating displaces the water molecules from the vicinity of the Au–Fe_3_O_4_ nanocomposite, leading to an increase in the MR values. However, at higher concentrations, the MR values are comparable and the PLL-coated Au–Fe_3_O_4_ NPs would be ideal for cancer imaging due to the higher bioavailability and minimal cytotoxicity. These results indicated that the formulated nanocomposites are superparamagnetic and ideal for cancer MR imaging.

### Cytotoxicity study by PLL–Au– Fe_3_O_4_ NPs and its intracellular uptake

**Fig. 4:**
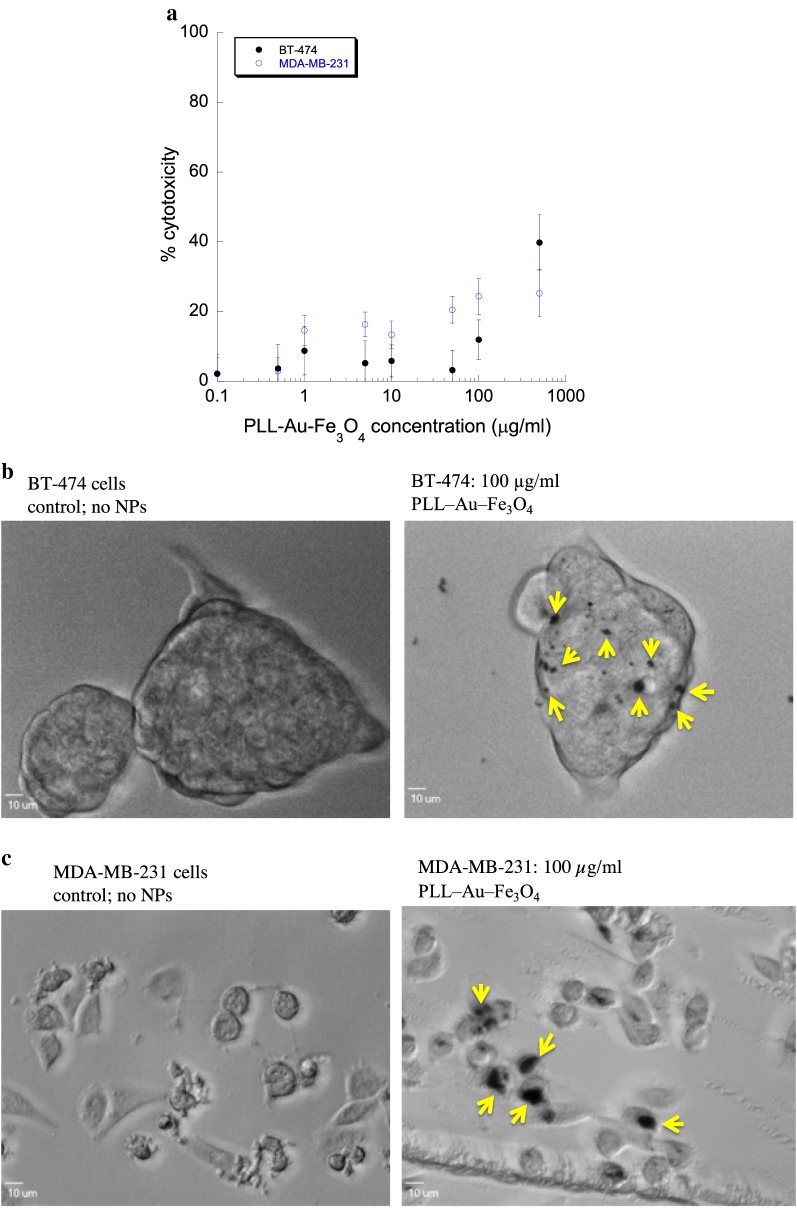
**a** Cytotoxicity of PLL–Au–Fe_3_O_4_ NPs in BT-474 and MDA-MB-231 cells using live-dead assay at increasing NP concentration; Cellular uptake of PLL–Au–Fe_3_O_4_ NPs in **b** BT-474 and **c** MDA-MB-231 cells. Arrows indicate the NPs inside cells. Cells without any NP treatment (control) did not show the black NP dots inside cells. Scale bar = 10 μm

Figure 4a shows the cytotoxicity induced by various concentrations of PLL–Au–Fe_3_O_4_ NPs in BT-474 and MDA-MB-231 cells is dose-dependent. PLL–Au–Fe_3_O_4_ NPs did not show much cytotoxicity (≤ 20% cell death) at a concentration ≤ 100 μg/ml, and hence the concentration was chosen for subsequent studies. To investigate the intracellular uptake of the NPs, phase contrast images were used. It can be seen from Fig. 4b and c that the PLL–Au–Fe_3_O_4_ NPs (black spots as indicated by arrows) entered in the cytoplasm of BT-474 and MDA-MB-231 cells. No black clusters were found in control cells without any NPs indicating the internalization of NPs by the cells. No morphological changes in cellular physiology have been observed when compared to control cells demonstrating negligible cytotoxicity by PLL–Au–Fe_3_O_4_ NPs. The uptake of PLL–Au–Fe_3_O_4_ NPs was further evaluated by TEM imaging (Additional file 1: Figure S4). Clearly, cells treated with NPs show a remarkable distribution inside endoplasmic vesicles of the cells.

### Photothermal effects by PLL–Au–Fe_3_O_4_

**Fig. 5:**
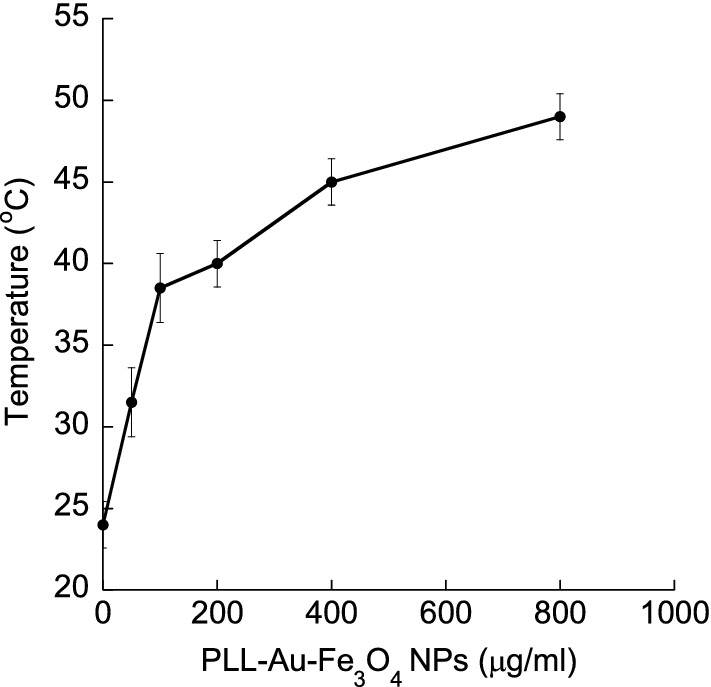
Temperature change of a PBS solution containing PLL–Au–Fe_3_O_4_ NPs under an 808 nm laser irradiation as a function of different NP concentrations

**Fig. 6:**
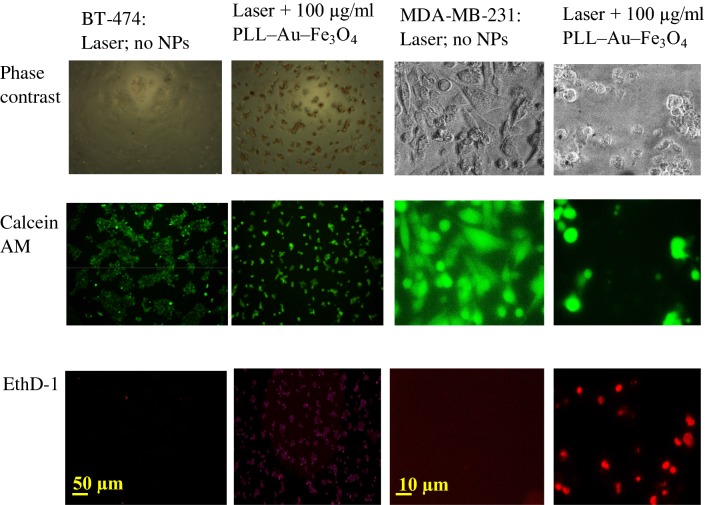
Phase contrast (row 1) and fluorescence (rows 2 and 3) images of BT-474 and MDA-MB-231 cells treated with 2 h incubation of PLL–Au–Fe_3_O_4_ followed by 10 min laser irradiation and 72 h of incubation in the medium. Uninternalized NPs were washed with PBS before the imaging. Green fluorescence represents live cells as stained with calcein AM, while the red fluorescence represents dead cells as stained with EthD-1. Scale bars are shown on images

**Fig. 7:**
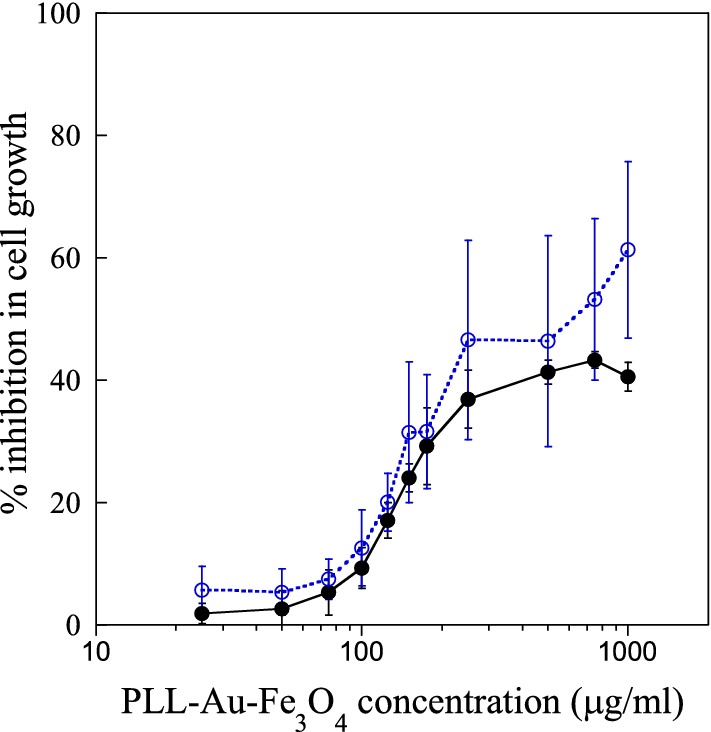
Quantitative assay of BT-474 (solid line; filled circle) and MDA-MB-231 (dotted line; open circle) cell growth inhibition following photothermal therapy in presence of PBS + laser and 100 μg/ml of PLL–Au–Fe_3_O_4_ NPs. The data represent the average of three independent experiments

In order to investigate, the potential of PLL–Au–Fe_3_O_4_ as nanothermal ablators, suspensions of the NPs in PBS were irradiated using 808 nm light emitted by a laser. The photothermal behavior of PLL–Au–Fe_3_O_4_ NPs was investigated by a temperature change versus the NP concentration (Fig. 5). Clearly, the NPs were able to induce a temperature enhancement in a concentration-dependent manner. The temperature of the NP suspension reached 50 °C at the PLL–Au–Fe_3_O_4_ NP concentration of 800 μg/ml. Laser irradiation of PBS negative control without NPs did not show any temperature increase under the same conditions. The results indicate that the synthesized PLL–Au–Fe_3_O_4_ NPs were able to transform NIR laser into heat under laser irradiation. For laser treatment, BT-474 and MDA-MB-231 cells were first incubated with PLL–Au–Fe_3_O_4_ NPs for 2 h followed by a wash process to remove un-internalized NPs, laser irradiation for 10 min and then for additional 72 h incubation period. In the control groups (cells + laser irradiation in absence of NPs), no significant cell death was observed. The cells treated with 100 μg/ml of PLL–Au–Fe_3_O_4_ NPs but without laser illumination reached 90 ± 4.7% of cell survival rate compared to those treated with PBS control. In contrast, the combination of PLL–Au–Fe_3_O_4_ NPs and laser treated cells underwent photothermal destruction as shown by both phase contrast and cell viability (green live cells) and dead cell (red stain) staining (Fig. 6). The groups for PLL–Au–Fe_3_O_4_ NPs + 10 min exposure to laser illumination at 1 W/cm^2^ showed the inhibition in cell growth by 49 ± 12% in BT-474 cells (Fig. 7; filled circle) and 60 ± 10% in MDA-MB-231 cells (Fig. 7; open circle) suggesting significant therapeutic effects following the photothermal treatment. Cells without any treatment, with laser treatment alone and with NP groups showed ≤ 5% inhibition in cell growth. The cytocompatibility of PLL–Au–Fe_3_O_4_ NPs was further assessed by MTT cell viability assay (Additional file 1: Figure S5). The quantitative analysis further reveals that the percentage of cell viability decreased to 63.5 ± 5.5% and 31.6 ± 3.2% in BT-474 and MDA-MB-231 cells, respectively at concentrations ≥ 10 μg/ml of NPs following laser irradiation.

## Discussion

A dumbbell-shaped Au–Fe_3_O_4_ superparamagnetic NPs is synthesized that is similar in morphology to multifaceted Janus particles [39, 40]. The structure (Au–decorated Fe_3_O_4_) is chosen to provide added advantages of both Au and Fe_3_O_4_ functionalities for the full utilization of their potential. Several Au dots on the Fe_3_O_4_ host ensures that there is a maximum number of spots for molecular recognition since Au-terminal acts as anchors for targeting agents. Perhaps the most challenging aspect of applying Au–Fe_3_O_4_ NPs involves the colloidal stability and shelf-life, surface functionalization, cellular internalization, and biocompatibility. Designing a properly grafted surface is therefore of paramount importance. Dextran [41], PEG [42], and silica coating [43] have been used extensively, however, each method has its benefits and drawbacks and requires trade-offs and optimizations. For example, although silica coatings can provide colloidal stability, it may also retard R_2_ relaxation in MRI imaging, as well dampen the superparamagnetic moment which reduces hyperthermia efficiency. Reduction in the superparamagnetic moment also leads to the necessity for increased dosage of the magnetic NPs. In that regards, the Janus-like particle with larger Fe_3_O_4_ anchored with several Au dots serves the multifaceted purpose of retaining high superparamagnetic moment over larger size [35] as well as optimizing SPR and photothermal effect of Au. Here, we report a simple and versatile method to adsorb PLL on bare Au–Fe_3_O_4_ dispersed in aqueous solutions. The polycationic PLL backbone ensures tight coulomb attachment onto anionic surfaces of Au endowing the NP surface with a robust coverage.

This approach can be generalized to many anionic surfaces onto which cationic polymers can adhere tightly, such as glass or silica. The results are comparable with previous studies on Au nanoshells where polymers (i.e., polyethylene imine; PEI) have been used as an intermediate separating layer during the fabrication of Au–Fe_3_O_4_ where the polymer layer prevents the migration of Au shell into the magnetic core by connecting between the core and shell structure [44, 45]. In contrast, we show an outer layer of PLL on the surface of Au–Fe_3_O_4_ that attribute to the columbic interactions between the Au surface and aliphatic monoamines that is clearly responsible for the observed stability of Au–Fe_3_O_4_ against aggregation in PBS. The TEM images showed the presence of a layer of PLL of approximately 9 nm thickness. The PLL coating improved the interaction of negatively charged Au–Fe_3_O_4_ at the cellular level by the resulting surface charge as confirmed by the zeta potential data.

The next step of PLL–Au–Fe_3_O_4_ involves cell culture experiments that suggest the overall intracellular uptake of the NPs at a non-toxic dose in breast cancer cells. The live/dead assay indicates that PLL–Au–Fe_3_O_4_ NPs are not cytotoxic in breast cancer cells at concentrations ≤ 100 μg/ml (Fig. 5). When compared to other cell viability data of polymer-coated NPs, our results are comparable to the literature data showing 20% reduction in cell viability using similar NP concentrations [45, 46]. Furthermore, the results from the phase contrast imaging microscopy demonstrated the effective internalization of the NPs presumably due to the combination of electrostatic interaction with the negatively charged glycocalyx on the cell membrane [47, 48] and the receptor-mediated endocytosis via EGCG [49–53]. The results are in agreement with the previous finding where Au NPs functionalized with methoxy-PEG-thiol were internalized by MDA-MB-231 cells via endocytosis [54, 55]. Importantly, the observed uptake of PLL–Au–Fe_3_O_4_ NPs by breast cancer cells associated with low toxicity suggest a potential theranostic use of these Au NPs. It is worth noting that PLL–Au–Fe_3_O_4_ had higher levels of uptake in MDA-MB-231 cells than BT-474 cells. The differences in cell surface protein expressions and proteoglycans may attribute to the differential uptake of NPs inside the cells [56–58]. Importantly, the higher observed uptake of PLL–Au–Fe_3_O_4_ in MDA-MB-231 than BT-474 cells was associated with pronounced toxicity (Fig. 5 and 6) suggesting a potential theranostic use of these gold nanoparticles.

Multiple studies have demonstrated the heating effect of Au shell NPs. Thermal ablation using PEG-coated Au nanoshells with silica cores have been reported to induce photothermal morbidity in SK-BR-3 breast cancer cells [59]. The treated cells revealed lost in cell membrane integrity and cell death that was not observed in the controls with laser irradiation or gold nanoshells alone [59]. Photothermal destruction was effective in reducing colon tumors in mice with a mean survival life of 90 days after the treatment [60]. We found that at 100 μg/ml of PLL–Au–Fe_3_O_4_ and 10 min laser exposure, a ΔT of 15 ± 7.6 °C was achieved. The current study reveals the effectiveness of using PLL–Au–Fe_3_O_4_ as a potent MRI-visible photosensitizer during the laser irradiation of cancer cells [45, 61, 62]. The noticeable combination of properties of PLL–Au–Fe_3_O_4_ with the local application of NIR laser irradiation offers a promising approach for the photothermal ablation therapy of breast cancer cells following internalization inside cells.

## Conclusions

A multifunctional NP was constructed using the PLL polymer coating on Au–Fe_3_O_4_ with a mean diameter of ~ 60 nm that are less prone to aggregation and thus suitable for a wide range of biological applications. PLL–Au–Fe_3_O_4_ NPs showed good colloidal stability, NIR light absorption property, magnetic relaxivity and cytocompatibility. Upon exposure to a NIR light, the SPR of Au increased their temperature inside the cells. The PLL–Au–Fe_3_O_4_ NPs were also demonstrated the importance of having a surface of the polymer layer that enhanced MRI contrast. As an integrated all-in-one NP platform, the polymeric theranostic agent was shown for intracellular uptake and hyperthermal treatments of breast cancer cells for further translational therapeutic applications.

## Additional files

**Additional file 1: Figure S1.** **(a)** PLL trypan blue standard curve; and **(b)** Au–Fe3O4 concentration measurement standard line. **Figure S2.** Hydrodynamic diameters of **(a)** Au–Fe3O4 NPs and **(b)** PLL coated Au–Fe3O4 NPs in water as measured by dynamic light scattering (DLS). Three color codes indicate three independent DLS measurements. **Figure S3.** Magnetic relaxation (MR) characterization of **(A and C)** Au–Fe3O4 and **(Band D)** PLL–Au–Fe3O4 NPs. **A)** Spin-spin MR (T2 ms) of bare Au–Fe3O4 NPs was found to be 190 ms, whereas, it was **B)** 280 ms for PLL–Au–Fe3O4 NPs at a given concentration of 10 μg/mL. This change in T2 MR value is the indicative of effective PLL coatings. Concentration dependent (1–100 μg/mL) T2 values were obtained in PBS (pH 7.2) and in 10% FBS for **C)** Au–Fe3O4 and **D)** PLL–Au–Fe3O4 NPs. These results indicated that with increase in concentration, the MR properties of these NPs increase and were found to be stable in physiological pH and in sera. **Figure S4.** The PLL–Au–Fe3O4 NPs uptake by **(a)** BT-474 and **(b)** MDA-MB-231 cells after 24 h incubation. Red circles indicate the endosomal vesicles that clearly retain the NPs as appeared in dark black spots. **Figure S5.** MTT cell viability assay of BT-474 (filled circle, solid line) and MDA-MB-231 (open circle, dotted line) cell viability after treatment with PLL–Au–Fe3O4 NPs under a 650 nm laser irradiation for 10 min. **Table S1.** The percentage encapsulation efficiency of PLL on Au–Fe3O4 NPs.

**Additional file 2: Movie S1.** This video shows Au–Fe3O4 (left) and PLL–Au–Fe3O4 NPs (right) where PLL coating prevents NP settle down in water and therefore black PLL–Au–Fe3O4 NPs’ colloidal suspension is observed.
