# Supplementary material for: Polymer coated gold-ferric oxide superparamagnetic nanoparticles for theranostic applications
Source: J Nanobiotechnology. 2018 Oct 13;16:80. doi: 10.1186/s12951-018-0405-7 (PMC6186064; doi:10.1186/s12951-018-0405-7)
Supplement: Supplementary file 1 — Additional file 1: Figure S1. (a) PLL trypan blue standard curve; and (b) Au–Fe3O4 concentration measurement standard line. Figure S2. Hydrodynamic diameters of (a) Au–Fe3O4 NPs and (b) PLL coated Au–Fe3O4 NPs in water as measured by dynamic light scattering (DLS). Three color codes indicate three independent DLS measurements. Figure S3. Magnetic relaxation (MR) characterization of (A and C) Au–Fe3O4 and (Band D) PLL–Au–Fe3O4 NPs. A) Spin-spin MR (T2 ms) of bare Au–Fe3O4 NPs was found to be 190 ms, whereas, it was B) 280 ms for PLL–Au–Fe3O4 NPs at a given concentration of 10 μg/mL. This change in T2 MR value is the indicative of effective PLL coatings. Concentration dependent (1–100 μg/mL) T2 values were obtained in PBS (pH 7.2) and in 10% FBS for C) Au–Fe3O4 and D) PLL–Au–Fe3O4 NPs. These results indicated that with increase in concentration, the MR properties of these NPs increase and were found to be stable in physiological pH and in sera. Figure S4. The PLL–Au–Fe3O4 NPs uptake by (a) BT-474 and (b) MDA-MB-231 cells after 24 h incubation. Red circles indicate the endosomal vesicles that clearly retain the NPs as appeared in dark black spots. Figure S5. MTT cell viability assay of BT-474 (filled circle, solid line) and MDA-MB-231 (open circle, dotted line) cell viability after treatment with PLL–Au–Fe3O4 NPs under a 650 nm laser irradiation for 10 min. Table S1. The percentage encapsulation efficiency of PLL on Au–Fe3O4 NPs. [file 12951_2018_405_MOESM1_ESM.pdf]

# Additional e-file 1

# Polymer Coated Gold-Ferric Oxide Superparamagnetic Nanoparticles for Theranostic Applications

Muhammad Raisul Abedin,[a] Siddesh Umapathi,[b] Harika Mahendrakar,[a] Tunyaboon Laemthong,[a] Holly Coleman,[b] Denise Muchangi,[c] Santimukul Santra,[c] Manashi Nath,[b] and Sutapa Barua[a,1]

[a] Department of Chemical and Biochemical Engineering

Missouri University of Science and Technology, Rolla, MO 65409

[b] Department of Chemistry

Missouri University of Science and Technology, Rolla, MO 65409

[c] Department of Chemistry

Pittsburg State University, Pittsburg, KS 66762

---

[1] To whom correspondence should be addressed. Email: baruas@mst.edu. Department of Chemical & Biochemical Engineering, 110 Bertelsmeyer Hall, 1101 N. State Street, Rolla, MO 65409-1230

(a)

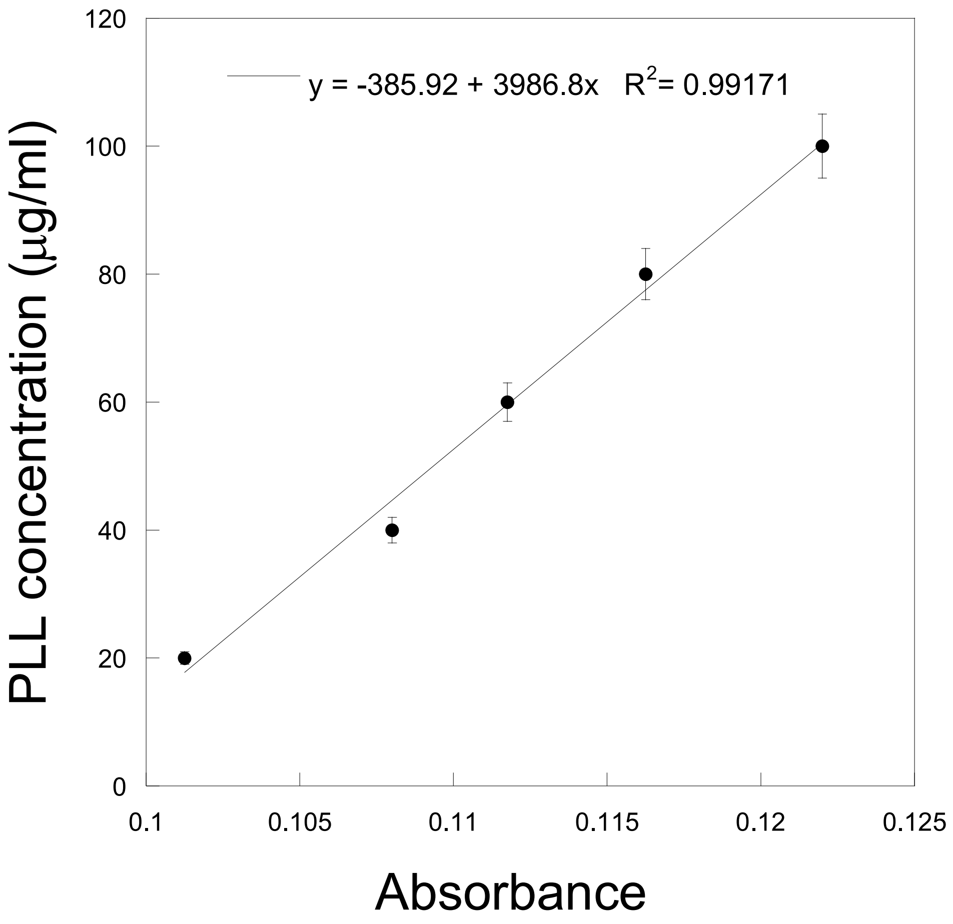

y = -385.92 + 3986.8x R2= 0.99171
PLL concentration (µg/ml)
Absorbance
0
20
40
60
80
100
120
0.1
0.105
0.11
0.115
0.12
0.125

**(b)**

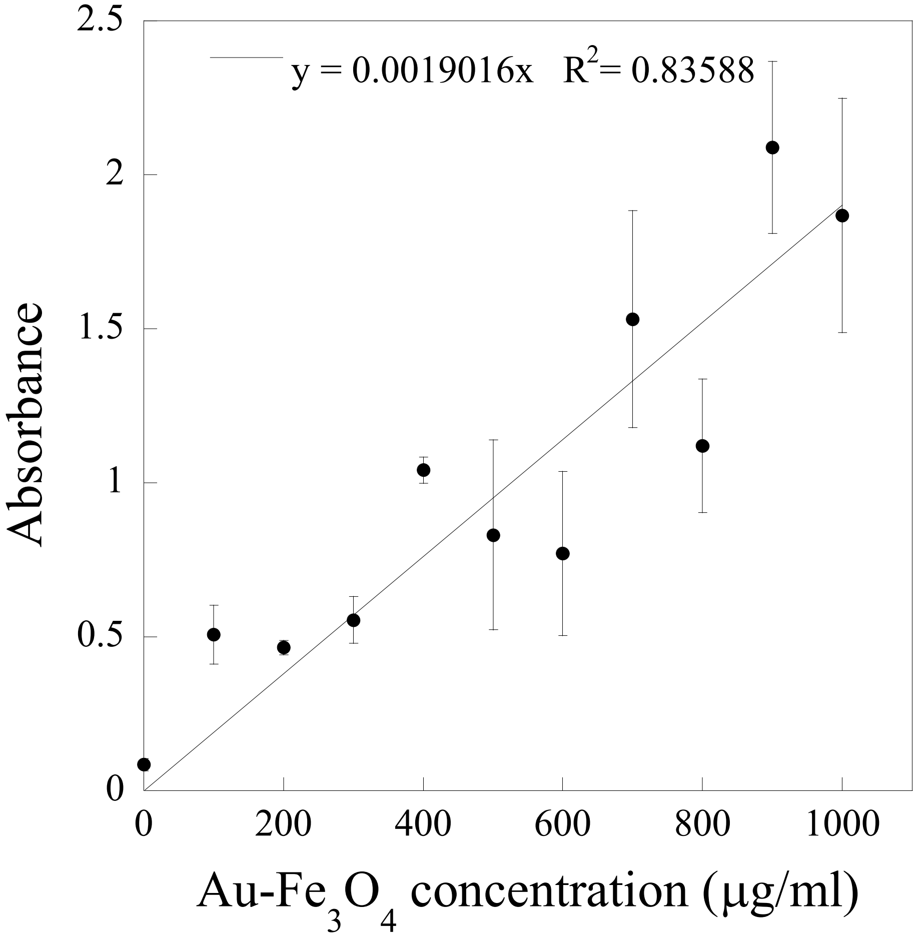


**Figure S1.** **(a)** PLL trypan blue standard curve; and **(b)** Au–$Fe_3O_4$ concentration measurement standard line

**(a)**

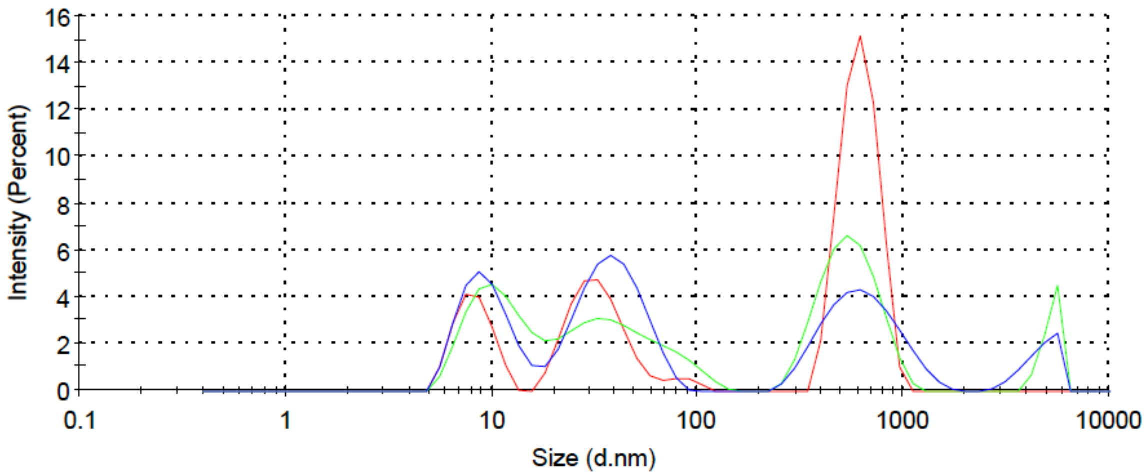


**(b)**

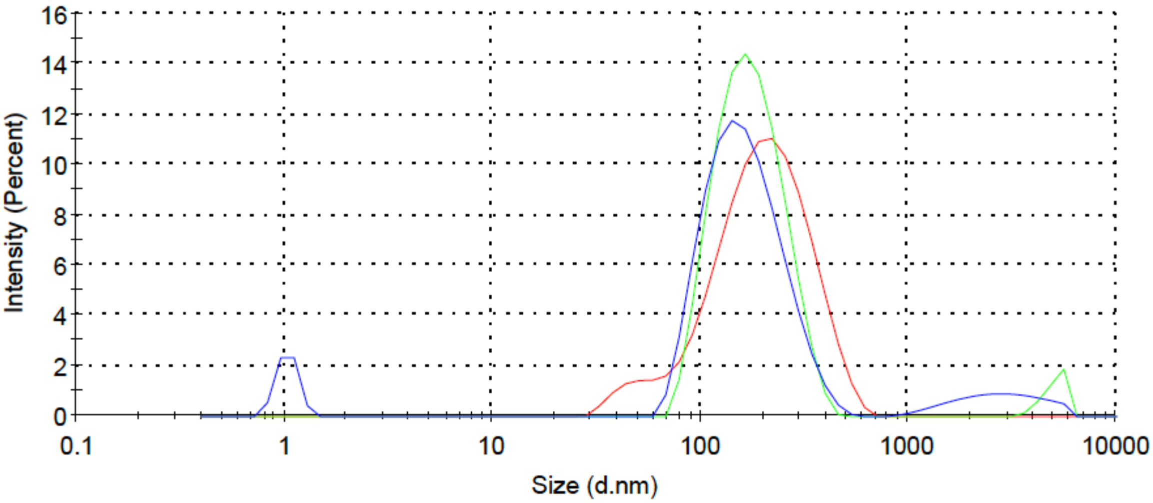


**Figure S2.** Hydrodynamic diameters of **(a)** Au–$Fe_3O_4$ NPs and **(b)** PLL coated Au–$Fe_3O_4$ NPs in water as measured by dynamic light scattering (DLS). Three color codes indicate three independent DLS measurements.

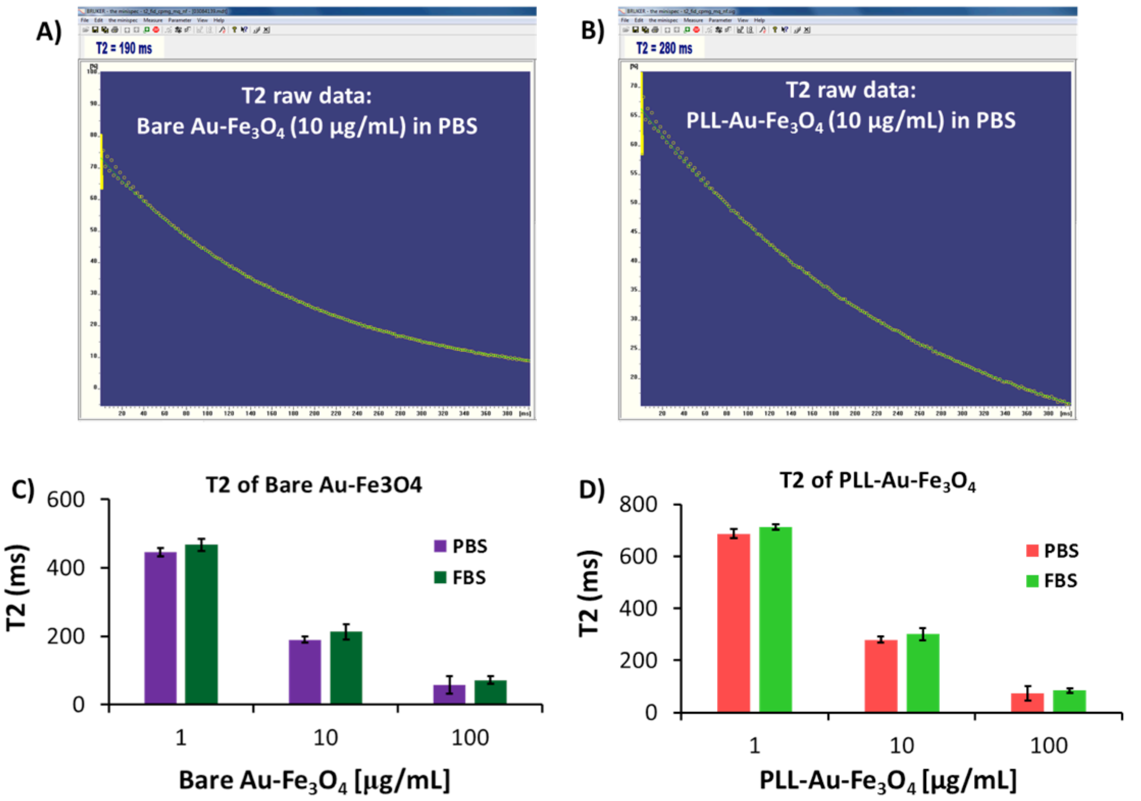


**Figure S3.** Magnetic relaxation (MR) characterization of **(A and C)** Au–$Fe_3O_4$ and **(B and D)** PLL–Au–$Fe_3O_4$ NPs. **A)** Spin-spin MR ($T_2$ ms) of bare Au–$Fe_3O_4$ NPs was found to be 190 ms, whereas, it was **B)** 280 ms for PLL–Au–$Fe_3O_4$ NPs at a given concentration of 10 μg/mL. This change in $T_2$ MR value is the indicative of effective PLL coatings.

Concentration dependent (1-100 μg/mL) $T_2$ values were obtained in PBS (pH 7.2) and in 10% FBS for **C)** Au–$Fe_3O_4$ and **D)** PLL–Au–$Fe_3O_4$ NPs. These results indicated that with increase in concentration, the MR properties of these NPs increase and were found to be stable in physiological pH and in sera.

**(a)**

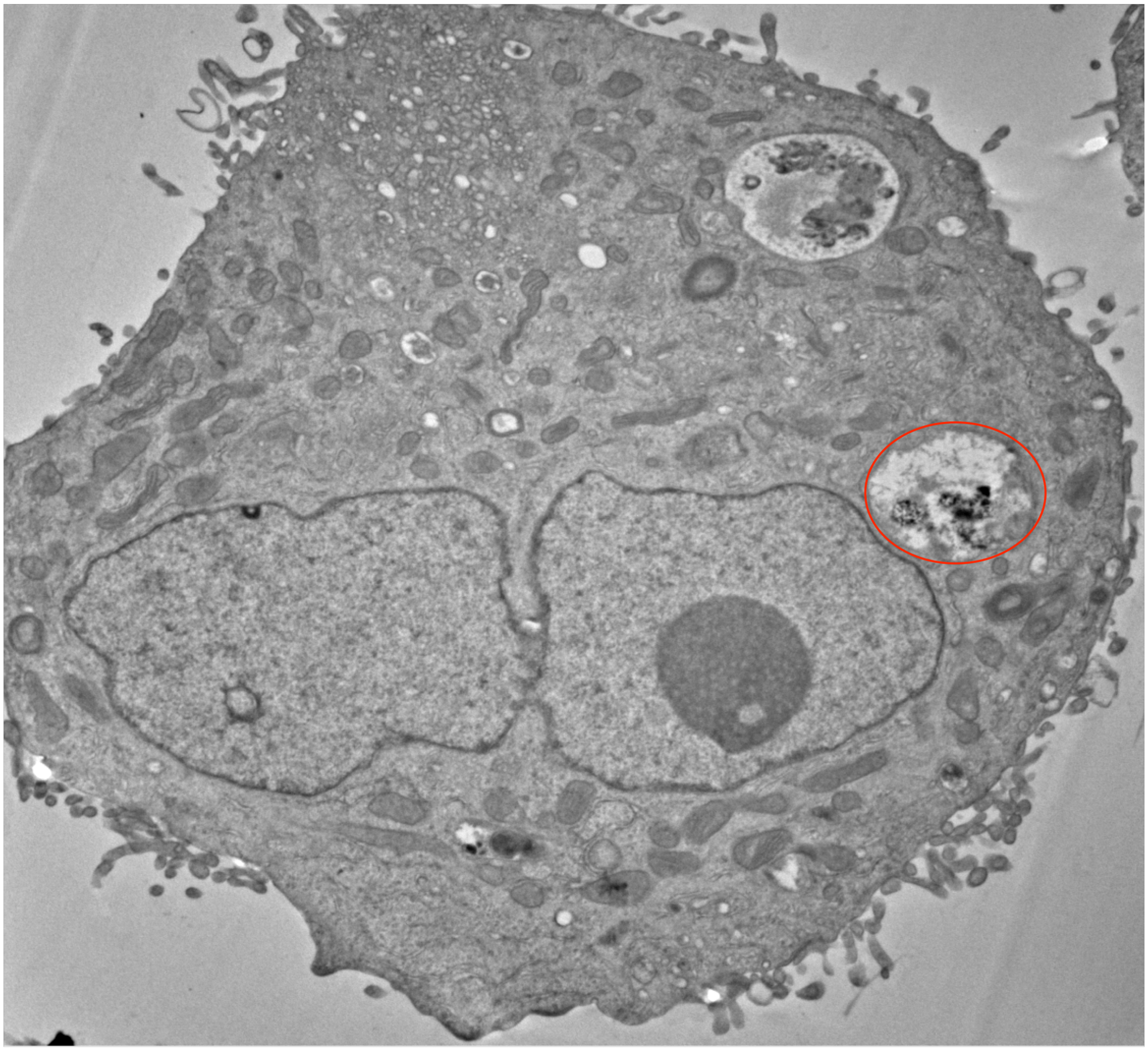

**(b)**

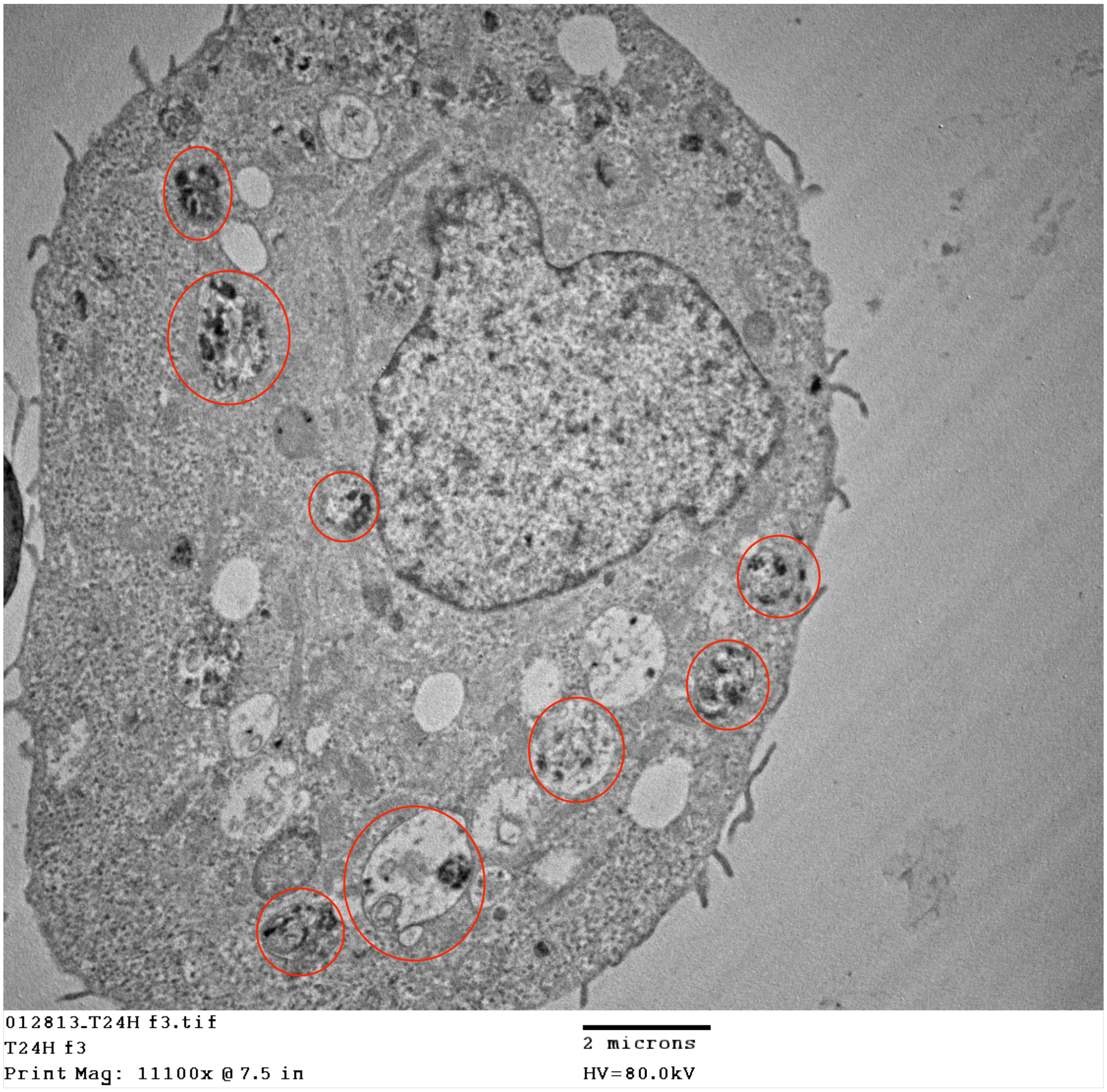


**Figure S4.** The PLL–Au–$Fe_3O_4$ NPs uptake by **(a)** BT-474 and **(b)** MDA-MB-231 cells after 24 h incubation. Red circles indicate the endosomal vesicles that clearly retain the NPs as appeared in dark black spots.

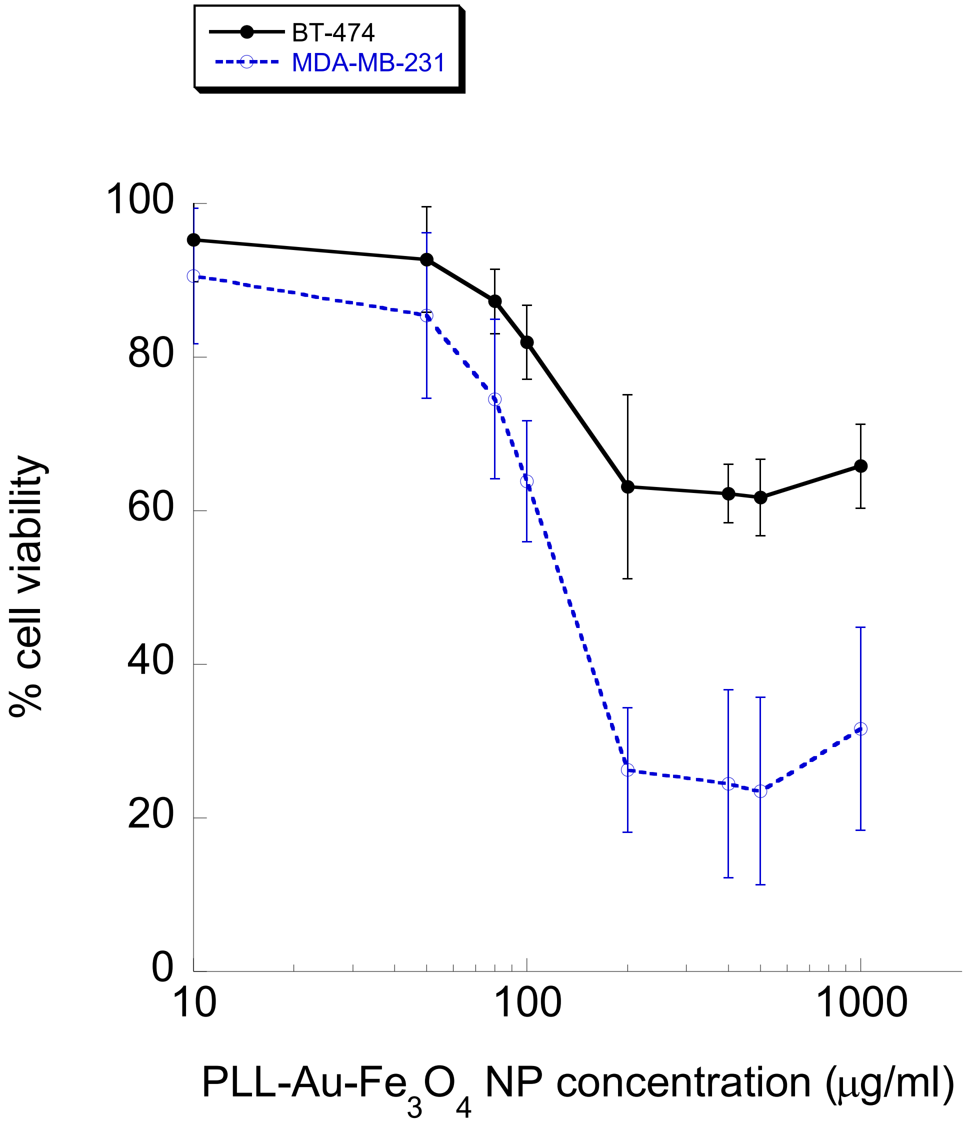


**Figure S5.** MTT cell viability assay of BT-474 (filled circle, solid line) and MDA-MB-231 (open circle, dotted line) cell viability after treatment with PLL–Au–$Fe_3O_4$ NPs under a 650 nm laser irradiation for 10 min.

**Table S1.** The percentage encapsulation efficiency of PLL on Au–$Fe_3O_4$ NPs

| mg Au–$Fe_3O_4$ | mg of PLL in PLL–Au–$Fe_3O_4$ | % PLL encapsulation efficiency |
|---|---|---|
| 25 | 0.47 ± 0.03 | 73 ± 3 |
